# The Association between Arrhythmia and *Helicobacter pylori* Infection: A Meta-Analysis of Case-Control Studies

**DOI:** 10.3390/ijerph13111139

**Published:** 2016-11-16

**Authors:** Jin Yan, Qiang She, Yifeng Zhang, Chang Cui, Guoxin Zhang

**Affiliations:** 1 Department of Gastroenterology, The First Affiliated Hospital with Nanjing Medical University, Nanjing 210029, China; yanjinnmu@163.com (J.Y.); zhangyifengyj@sina.com (Y.Z.); 2 The First Clinical Medical College, Nanjing Medical University, Nanjing 210029, China; sheqiangyanjin@sina.com (Q.S.); cuichangyanjin@sina.com (C.C.); 3 Department of Gastroenterology, Yangzhou NO.1 People’s Hospital, Yangzhou 225001, China; 4 Department of Cardiology, The First Affiliated Hospital with Nanjing Medical University, Nanjing 210029, China

**Keywords:** *Helicobacter pylori*, arrhythmia, meta-analysis

## Abstract

Arrhythmia is a common disease around the world and *Helicobacter pylori* (*H. pylori*) is a bacterium infecting 28% to 84% of subjects, depending on the population tested. However, the implication of *H. pylori* in cardiac arrhythmia is poorly understood. We performed this meta-analysis with an aim to identify the association between arrhythmia and *H. pylori*. We searched PubMed, Embase, Web of Science, and the Cochrane library databases to select studies on the association between arrhythmia and *H. pylori*. In the arrhythmia group, 392 (58.1%) were *H. pylori*-positive and in the control group 640 (47.8%) were *H. pylori*-positive. Compared to the controls, the infection rate of *H. pylori* was higher in patients with arrhythmia than in controls (odds ratio (OR) = 1.797, 95% confidence interval (CI): 1.081–2.988, *p* < 0.05). Subgroup analysis indicated that *H. pylori* infection was a risk factor for atrial fibrillation in Asia and Africa. Therefore, a correlation between *H. pylori* infection and arrhythmia may exist and *H. pylori* eradication may decrease the occurrence of arrhythmia, especially in Asia and Africa.

## 1. Introduction

Arrhythmias are common and are increasing, particularly atrial fibrillation. Arrhythmias may be divided into different categories based on various criteria. For instance, based on the beating rate, arrhythmias include bradyarrhythmia and tachyarrhythmia. In addition, arrhythmias can be divided into supraventricular and ventricular arrhythmias depending on the pathogenic area [1]. The lifetime risk for the development of atrial fibrillation is about one in four for men and women over 40 years old [2]. The symptoms associated with arrhythmias include chest distress, chest pain, and headache. These arrhythmias may be associated with a good long-term prognosis but also may be associated with the risk of sudden death [2,3]. Previously, several studies have been conducted to elucidate the mechanisms of cardiac arrhythmias, and conventional theories mainly focus on the issues related to the formation and conduction of cardiac electrical impulse [1]. However, the exact causes are not clear and *Helicobacter pylori* (*H. pylori*) infection has been noted to be associated in previous studies. Therefore, we aimed to analyze the relationship within *H. pylori* infection and arrhythmia.

Recent studies have suggested that some microorganisms’ infection could contribute to the development of arrhythmia, including atrial fibrillation, through the inflammation cascade [4]. *H. pylori* is a Gram-negative microaerophilic bacterium and has been identified as the main pathogen for gastritis, peptic ulcer, and gastric cancer [5,6,7]. Besides gastroenterological disease, *H. pylori* is also found to be associated with arrhythmia. In 2005, Montenero et al. revealed for the first time a highly significant correlation between *H. pylori* infection and atrial fibrillation [8]. Other groups also showed that Cag-A positive *H. pylori* strains were related to idiopathic dysrhythmias [9]. However, Platonov et al. have reported no significant association between atrial fibrillation and *H. pylori* infection [10]. Thus, whether or not *H. pylori* is implicated in the pathogenesis of arrhythmia is inconclusive. 

We conducted this meta-analysis to obtain a more comprehensive estimate of the association between arrhythmia and *H. pylori*. The findings strengthening the association between arrhythmia and *H. pylori* infection could provide a new approach for treatment of stubborn arrhythmia.

## 2. Materials and Methods

### 2.1. Data Sources and Search Strategy

The meta-analysis was carried out according to the guidelines of the PRISMA statement and MOOSE [11,12]. We searched PubMed, Embase, Web of Science, and Cochrane library for eligible literature until May 2016. The medical search heading (MeSH) terms used in the search were “helicobacter pylori” combined with “arrhythmias”. The electronic search strategy for PubMed was (“helicobacter pylori”(MeSH Terms) OR (“helicobacter”(All Fields) AND “pylori”(All Fields)) OR “helicobacter pylori”(All Fields)) AND (“arrhythmias, cardiac”(MeSH Terms) OR (“arrhythmias”(All Fields) AND “cardiac”(All Fields)) OR “cardiac arrhythmias”(All Fields) OR “arrhythmias”(All Fields)). In addition, we manually searched the reference lists of eligible studies identified from the databases. Excel and Endnote X7 software were used to organize the retrieved literature. 

### 2.2. Inclusion and Exclusion Criteria

Two reviewers (Jin Yan and Qiang She) checked the abstracts of the studies and read the full-texts if necessary to identify the final included studies. When disagreement appeared, we discussed and consulted the third reviewer (Yifeng Zhang). In addition, we turned to the original authors for more detailed data if necessary. Eligible studies strictly met the following criteria: (1) the studies utilized a case-control, prospective, or cross-sectional study design; (2) the studies contained sufficient data for investigating the association between *H. pylori* infection and arrhythmia; (3) *H. pylori* infection was diagnosed through the urea breath test (UBT) and/or serology and/or histology and/or culture in both case and control groups; (4) the studies were published in English. Articles were excluded if they were any of the following: (1) review articles, meta-analyses, letters, commentaries, or abstracts presented in conferences; (2) duplicates or continued work of previous publications; (3) studies without complete data; (4) not in English.

### 2.3. Quality Assessment and Data Extraction

To ensure the quality of the meta-analysis, the Newcastle-Ottawa quality assessment scale (NOS) was used and each item in the NOS was assessed for all included studies. The final score ranged from zero to nine stars for each study and a score of five stars or more was regarded to be optional [13,14]. Two authors (Jin Yan and Qiang She) independently assessed the quality of each study, and any disagreement was resolved through a discussion with the third author (Yifeng Zhang).

After quality assessments, all authors extracted information from each paper and organized the data in Excel (Microsoft, Redmond, WA, USA) independently. The following data were retrieved from each study: the first author, the year of publication, the country and continent, study design, study size, type of arrhythmia, diagnosis method for *H. pylori* infection detection, arrhythmia group with *H. pylori* (+/−), control group with *H. pylori* (+/−), clinical characteristics of the populations, and other substantial information.

### 2.4. Statistical Analysis

In this meta-analysis, we calculated dichotomous variables using the odds ratio (OR) with the 95% confidence interval (CI) to measure the strength of the association of *H. pylori* infection and arrhythmia. Heterogeneity was assessed by the test of inconsistency index (I^2^) and the Cochran-Q method. I^2^ value more than 50% or *p* value less than 0.1 suggested the presence of heterogeneity and the random effects model was applied. We also performed subgroup analyses to seek the sources of heterogeneity. In addition, sensitivity analysis was conducted to evaluate whether omitting one study in each turn substantially altered the main results. Finally, the publication bias was analyzed by the funnel plots (*p* value lower than 0.05 indicated a significant publication bias). Statistical analysis was conducted utilizing Stata 12.0 (Stata Corporation, College Station, TX, USA) software.

## 3. Results

### 3.1. Data Selection

**Figure 1:**
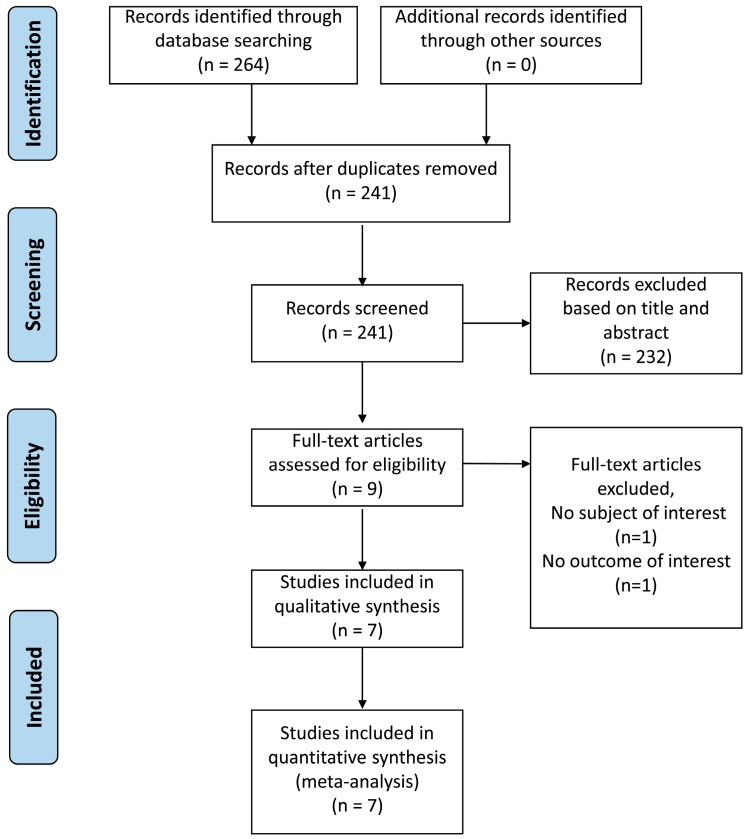
Flow diagram of the study selection process.

The review process is shown in Figure 1. The initial search revealed a total of 264 studies, including 23 duplicate hits. We then screened titles and abstracts, and excluded 232 papers, among which 43 were reviews, 186 were not relevant to this meta-analysis, and three were not in English. Finally, nine studies remained, whose full-text versions were retrieved and two of them were excluded. One was about the association of *Helicobacter cinaedi* with atrial arrhythmias, and in the other paper we failed to extract sufficient data [8,15]. Thus, a total of seven studies met the review criteria and were analyzed in our meta-analysis [9,10,16,17,18,19,20].

### 3.2. Study Characteristics and Quality Assessment

The eligible articles were published from 2006 to 2015 accumulating 675 arrhythmia patients and 1339 controls. All the included studies were conducted using a case-control study design. The studies’ characteristics are presented in Table 1, including the first author, publication year, country, continent, study design, study size, type of arrhythmia, diagnosis method for *H. pylori* infection, and the number of *H. pylori* positive and negative subjects in arrhythmia and control groups. Demographic characteristics including age and gender are shown in Table S1. In addition, seven studies were scored via NOS by two independent reviewers (J.Y. and S.M.) and scores were shown in Table 2.

### 3.3. Data Analysis

**Figure 2:**
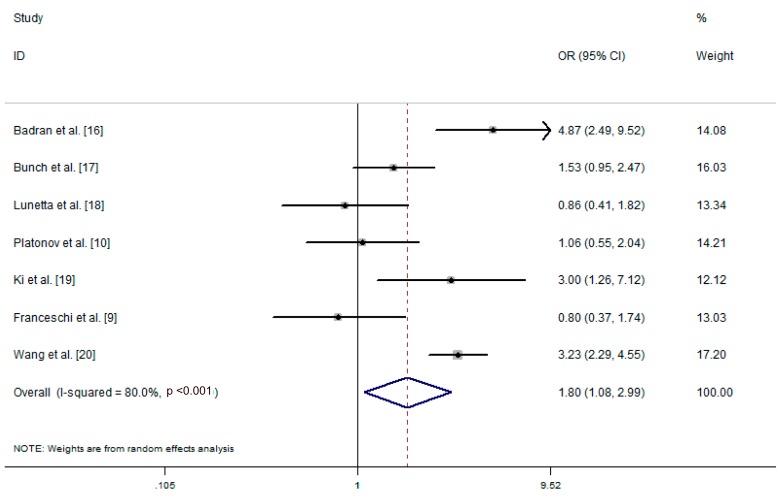
Effect estimates and pooled estimate of included studies reporting the association of arrhythmia and *Helicobacter pylori* infection. OR, odds ratio; CI, confidence interval.

In the seven eligible studies, 392/675 patients in the arrhythmia group were positive for *H. pylori* infection (58.1%), compared to 47.8% in the control group (640/1339). There was significant heterogeneity in the included studies (I^2^ = 80%, *p* < 0.001). Therefore, we used the random-effects model. The obtained pooled OR was 1.80 (95% CI: 1.08–2.99) (Figure 2) and the test for overall effect Z was 2.26 (*p* = 0.024). Thus, we conclude that there may be a relationship between *H. pylori* infection and risk of arrhythmia.

### 3.4. Subgroup Analysis and Sensitivity Analysis

**Figure 3:**
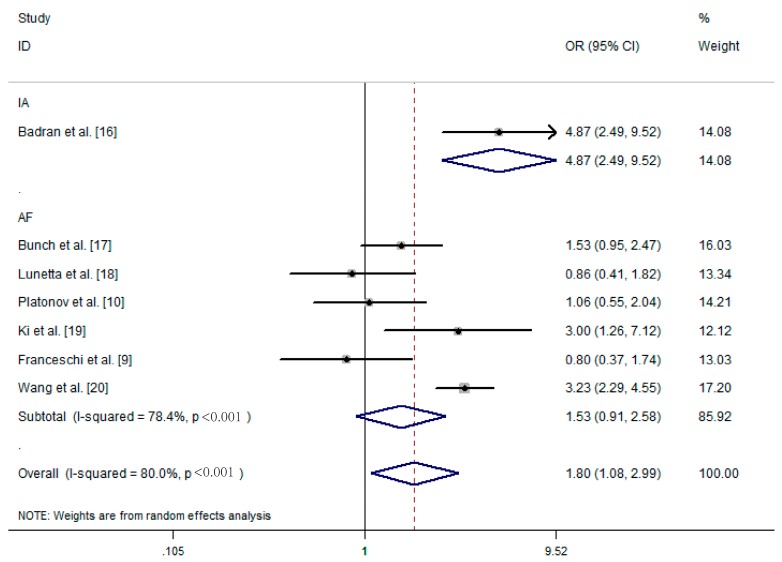
Forest plot showing the relationship between arrhythmia and *Helicobacter pylori* infection: studies divided into subgroups according to the type of arrhythmia. OR, odds ratio; CI, confidence interval.

**Figure 4:**
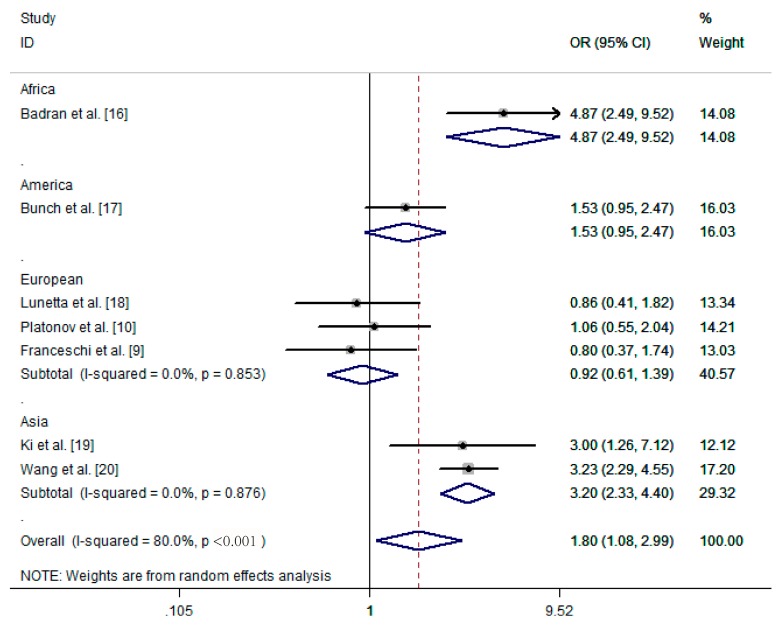
Forest plot showing the relationship between arrhythmia and *Helicobacter pylori* infection: studies divided into subgroups according to the continent of study origin. OR, odds ratio; CI, confidence interval.

**Figure 5:**
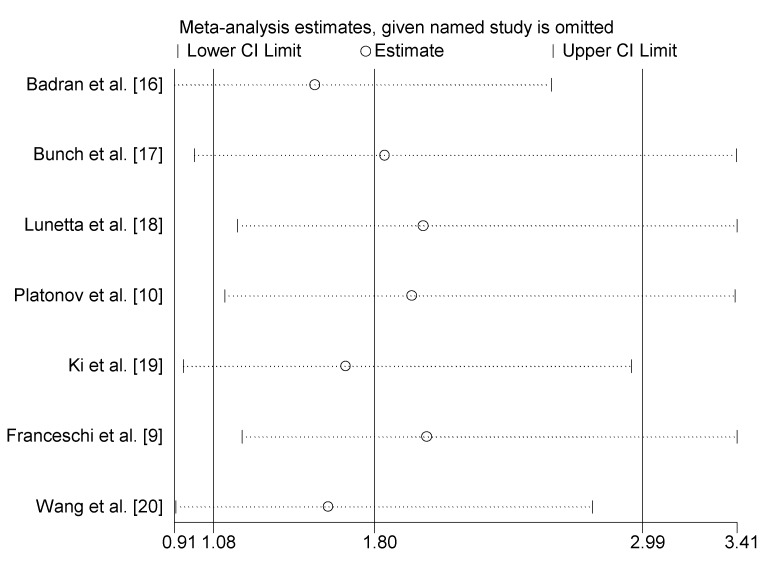
Sensitivity analysis of the meta-analysis.

Apart from using the random-effects model, we also performed subgroup analyses to analyze the source of heterogeneity. These seven studies focused on two kinds of arrhythmia, including idiopathic dysrhythmias and atrial fibrillation. Idiopathic dysrhythmias are defined as arrhythmias occurring in the absence of clinical evidence of organic cardiac diseases. As shown in Figure 3, *H. pylori* infection correlated with idiopathic dysrhythmias (OR = 4.87, 95% CI: 2.49–9.52). In the other six studies investigating the relationship between *H. pylori* infection and atrial fibrillation, the pooled OR was 1.53 (95% CI: 0.91–2.58, Z = 1.60, *p* = 0.111). We also found that the subgroup of the region played an important role in the heterogeneity of the results. Compared to those studies in America (OR = 1.53, 95% CI: 0.95–2.47, Z = 1.76, *p* = 0.079) and Europe (OR = 0.92, 95% CI: 0.61–1.39, Z = 0.41, *p* = 0.685), investigations in Asia (OR = 3.20, 95% CI: 2.33–4.40, Z = 7.15, *p* < 0.001) and Africa (OR = 4.87, 95% CI: 2.49–9.52, Z < 0.001) revealed that *H. pylori* was significantly related to atrial fibrillation. In addition, there was no significant heterogeneity in separated sub-groups (I^2^ = 0%, *p* > 0.1) (Figure 4). We further performed sensitivity analysis by removing one study at a time from our meta-analysis. None of the results were significantly altered (Figure 5), indicating that our results were robust. 

### 3.5. Publication Bias

**Figure 6:**
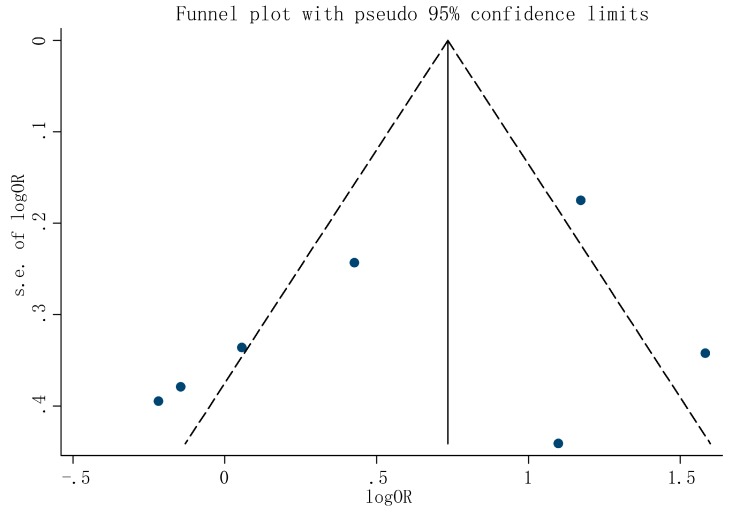
Funnel plot to explore publication bias.

To investigate the potential publication bias present in this study, the included studies were evaluated using Begg’s test. As shown in Figure 6, the distribution of studies on both sides was asymmetrical and the *p* value was 0.23. Thus, there was a publication bias in this meta-analysis.

## 4. Discussion

In this study, a total of seven case-control studies published between 2006 and 2015 that met our study criteria were included in this meta-analysis. We provided an overview of the relationship between *H. pylori* infection and arrhythmia with a total of 2014 subjects. Overall, the *H. pylori* infection rate in arrhythmia patients (58.1%, 392/675) was significantly higher than that in control individuals (47.8%, 640/1339) (OR = 1.797, 95% CI: 1.081–2.988), indicating that *H. pylori* may be related with arrhythmia. In these seven studies, six studies investigated the association between *H. pylori* infection and atrial fibrillation, a common type of arrhythmia. However, the result was not significant (OR = 1.53, 95% CI: 0.91–2.58). Then we divided the studies according to their origin and found that *H. pylori* infection was significantly associated with atrial fibrillation in Asia and Africa, but not in Europe and North America. This result suggested that *H. pylori* infection might play a role in the development of atrial fibrillation in the Asian and African population. 

Arrhythmia is a common disease that badly influences the quality of life [2]. Research on arrhythmia began in the last century, but the specific mechanism underlying the development of arrhythmia is still unclear [21]. It is well known that gastrointestinal disorders may share similar risk factors such as stress, smoking, and drinking with cardiac diseases. Disorders of digestive system and cardiovascular system usually co-exist and they also share similar symptoms, including chest pain and faintness [22,23]. In addition, some pathogens and biomolecules generated from myocardial inflammatory process were recognized to participate in arrhythmia generation [8]. Hence, many scientists have speculated that *H. pylori* infection can induce arrhythmia via activating inflammatory process. *H. pylori* is a bacterium that colonizes in the gastric epithelium and has been identified as a group 1 carcinogen for gastric cancer [24]. With the advances in the studies on *H. pylori* infection, the correlation between *H. pylori* and arrhythmia has begun to emerge, but the conclusion remains under debate. For example, in Italy, Montenero et al. have shown that atrial fibrillation significantly correlates with *H. pylori* infection; however, Platonov et al. did not find such a correlation [8,10]. In order to reveal the underlying mechanism, researchers have studied the correlation between *H. pylori* infection, inflammation, and arrhythmia, and they have found that tumor necrosis factor-α, interleukin-6 and C-reactive protein, which are markers reflecting the degree of inflammation, were closely related to the type and the duration of atrial fibrillation [25,26]. In addition, the antibody of *H. pylori* virulence factor cytotoxin-associated gene A showed ability to cross-react with the antigens of endothelial cell membranes [22]. On the other hand, *H. pylori* is both an inflammatory agent and an immune regulator, with virulence factor vacuolating cytotoxin A (VacA) efficiently blocking proliferation of T cells by inducing a G1/S cell cycle arrest [27,28,29]. *H. pylori* might modulate a T helper (Th) 1/Th2 balance and *H. pylori* VacA to induce Th1 suppression and might abrogate T regulatory cell functions, subsequently resulting in an increase in the risk of atrial fibrillation [29]. 

We also found heterogeneity in our analysis. Among these seven studies, six studies were about atrial fibrillation and one study investigated idiopathic dysrhythmias. The result for atrial fibrillation was not significant. Then, we also applied subgroup analysis based on origin and found that the continent where the study was conducted also presents a source of heterogeneity. The results were consistent with the different infection statuses of *H. pylori*. In Asia and Africa, the *H. pylori* infection rate was higher than that in western countries, which could be related to the different standards of hygiene and socioeconomics [30]. Therefore, our study detected a significant correlation between atrial fibrillation and *H. pylori* infection in Asia and Africa, but not in developed countries in Europe or North America.

Our meta-analysis also has some limitations. Firstly, all included studies were case-control studies and the quality of this type of study was not as high as that of randomized clinical trials. Secondly, the socioeconomic status of the subject has been identified as a high risk factor for *H. pylori* infection and therefore the control group should be socioeconomically matched to the arrhythmia group. Thirdly, when serological tests of *H. pylori* infection are applied, it was difficult to distinguish between past and active infection conditions. Fourthly, although we conducted an intense search in Pubmed, Embase, Web of Science, and the Cochrane Library, there might still be published studies in other databases that we missed. Also, we only reviewed papers written in English. A publication bias was found in the meta-analysis. Thus, a further systematic review extended to other languages should also be considered. In addition, some studies with negative results might not have been published, which could cause unavoidable publication bias in our study.

## 5. Conclusions

In conclusion, our study demonstrates that *H. pylori* should be considered as one of the risk factors for atrial fibrillation in Asia and Africa and may also be related to idiopathic dysrhythmia. However, large-scale and prospective studies examining the precise role of *H. pylori* in the development of arrhythmia will still be needed to further corroborate our conclusions. Our findings suggest that in Asia and Africa, eradication of *H. pylori* should be considered as an appropriate therapeutic regimen for some intractable atrial fibrillation cases.

## Figures and Tables

**Table 1:** Details of included studies reporting the association of *Helicobacter pylori* infection and arrhythmia.

Author/Year	Country/Continent	Study Design	Study Size	Type of Arrhythmia	Diagnostic Method for *H. pylori* Infection	Arrhythmia Group with *Hp* (+/−)	Control Group with *Hp* (+/−)
Badran et al. [16]	Egypt/Africa	Case-control study	162	IA	Serum *H. pylori* IgG antibodies	52/30	21/59
Bunch et al. [17]	USA/America	Case-control study	743	AF	Serum *H. pylori* IgG antibodies	54/29	362/298
Lunetta et al. [18]	Italy/European	Case-control study	180	AF	Serum *H. pylori* IgG antibodies	25/14	95/46
Platonov et al. [10]	Sweden/European	Case-control study	144	AF	Serum *H. pylori* IgG antibodies	41/31	40/32
Ki et al. [19]	Korea/Asia	Case-control study	96	AF	Serum *H. pylori* IgG antibodies	36/24	12/24
Franceschi et al. [9]	Italy/European	Case-control study	104	AF	13C urea breath test	23/31	24/26
Wang et al. [20]	China/Asia	Case-control study	585	AF	13C urea breath test	161/124	86/214

IA, idiopathic dysrhythmias; AF, atrial fibrillation.

**Table 2:** Results of quality assessment by Newcastle-Ottawa quality assessment scale (NOS) for case-control studies.

Study	Selection				Comparability	Exposure			Scores
	Is the Case Definition Adequate	Representativeness of Cases	Selection of Controls	Definition of Controls	Comparability of Cases and Controls on the Basis of the Design or Analysis #	Ascertainment of Exposure	Same Method of Ascertainment for Cases and Controls	Non-Response Rate	
Badran et al. [16]	*	*	*	*	**	*	*	-	8
Bunch et al. [17]	*	*	*	*	-	*	*	-	6
Lunetta et al. [18]	*	*	*	*	**	*	*	-	8
Platonov et al. [10]	*	*	*	*	**	*	*	-	8
Ki et al. [19]	*	*	-	*	-	*	*	-	5
Franceschi et al. [9]	*	*	*	*	**	*	*	-	8
Wang et al. [20]	*	*	*	*	**	*	*	-	8

# A maximum of two stars can be allotted in this category, one for age, the other for other controlled factors (gender, and so on); * One star; ** Two stars.

## Supplementary Materials

The following are available online at www.mdpi.com/1660-4601/13/11/1139/s1, Table S1: The demographic characteristics of the populations of the studies.

## Abbreviations

AF	atrial fibrillation
CI	confidence interval
I	inconsistency index
IA	idiopathic dysrhythmias
NG	not given
NOS	Newcastle-Ottawa quality assessment scale
OR	odds ratio
UBT	urea breath test
VacA	vacuolating cytotoxin A
