# Supplementary material for: The Association between Arrhythmia and Helicobacter pylori Infection: A Meta-Analysis of Case-Control Studies
Source: Int J Environ Res Public Health. 2016 Nov 16;13(11):1139. doi: 10.3390/ijerph13111139 (PMC5129349; doi:10.3390/ijerph13111139)
Supplement: Supplementary file 1 [file ijerph-13-01139-s001.pdf]

# Supplementary Materials: The Association between Arrhythmia and *Helicobacter pylori* Infection: A Meta-Analysis of Case-Control Studies

**Jin Yan, Qiang She, Yifeng Zhang, Chang Cui and Guoxin Zhang**

**Table S1.** The demographic characteristics of the populations of the studies.

| Author | Group | Mean Age (Year) | Gender (Proportion of Man) |
|---|---|---|---|
| Badran et al. [16] | AF | 62 ± 4 | 54.88% |
| | Control | 66 ± 3 | 60.00% |
| Bunch et al. [17] | AF | 70.9 ± 9.5 | 75.90% |
| | Control | 63.9 ± 10.7 | 76.67% |
| Lunetta et al. [18] | NG | NG | NG |
| | NG | NG | NG |
| Platonov et al. [10] | AF | 69.6 ± 8.3 | 68.06% |
| | Control | 69.8 ± 7.5 | 72.22% |
| Ki et al. [19] | AF | 61.28 ± 1.35 | 61.67% |
| | Control | 50.64 ± 2.18 | 41.67% |
| Franceschi et al. [9] | IA | 44 ± 17 | 74.07% |
| | Control | 45 ± 9 | 68.00% |
| Wang et al. [20] | AF | 63.8 ± 10.8 | 38.60% |
| | Control | 60.2 ± 10.65 | 51.52% |

AF, atrial fibrillation; IA, idiopathic dysrhythmias; NG, not given.
